# Slow wave sleep and accelerated forgetting

**DOI:** 10.1016/j.cortex.2016.08.013

**Published:** 2016-11

**Authors:** Kathryn E. Atherton, Anna C. Nobre, Alpar S. Lazar, Katharina Wulff, Roger G. Whittaker, Vandana Dhawan, Zsolt I. Lazar, Adam Z. Zeman, Christopher R. Butler

**Affiliations:** a Nuffield Department of Clinical Neurosciences, University of Oxford, Oxford, UK; b Oxford Centre for Human Brain Activity, University of Oxford, Oxford, UK; c John van Geest Centre for Brain Repair, Department of Clinical Neurosciences, University of Cambridge, UK; d Institute of Neuroscience, Newcastle University, Newcastle, UK; e Department of Physics, Babes-Bolyai University, Cluj-Napoca, Romania; f Cognitive and Behavioural Neurology, Peninsular Medical School, University of Exeter, UK

**Keywords:** Accelerated long-term forgetting, Transient epileptic amnesia, Memory, Consolidation, Slow wave sleep

## Abstract

We investigated whether the benefit of slow wave sleep (SWS) for memory consolidation typically observed in healthy individuals is disrupted in people with accelerated long-term forgetting (ALF) due to epilepsy. SWS is thought to play an active role in declarative memory in healthy individuals and, furthermore, electrographic epileptiform activity is often more prevalent during SWS than during wakefulness or other sleep stages. We studied the relationship between SWS and the benefit of sleep for memory retention using a word-pair associates task. In both the ALF and the healthy control groups, sleep conferred a memory benefit. However, the relationship between the amount of SWS and sleep-related memory benefits differed significantly between the groups. In healthy participants, the amount of SWS correlated positively with sleep-related memory benefits. In stark contrast, the more SWS, the smaller the sleep-related memory benefit in the ALF group. Therefore, contrary to its role in healthy people, SWS-associated brain activity appears to be deleterious for memory in patients with ALF.

## 1 Introduction

### 1.1 Accelerated long-term forgetting

Accelerated long-term forgetting (ALF) is a common symptom of transient epileptic amnesia (TEA) (Butler et al., 2007). People with ALF appear to learn and initially retain new information normally, but subsequently forget at an accelerated rate (Bell and Giovagnoli, 2007, Butler and Zeman, 2008). This phenomenon provides a novel opportunity to investigate post-encoding memory processes.

### 1.2 Sleep and memory in health

In healthy people, post-encoding sleep is thought to play an important role in memory. This role is not fully understood, but may have both passive and active components. Sleep provides a temporary shield from potentially interfering cognitive stimulation. Cognitive stimulation during a wakeful retention interval may cause forgetting through a number of mechanisms – interference with access at the point of retrieval (e.g., Brown, Neath, & Chater, 2007); disruption of the memory trace (e.g., Wixted, 2004, Wixted, 2010); and interference with certain consolidation processes (e.g., Mednick et al., 2011, Wixted, 2004) – and so some part of sleep's benefit for memory retention is likely due to protection from this retroactive interference.

In addition to this passive, or permissive, benefit afforded by sleep, slow wave sleep (SWS) is thought to play an active role in declarative memory. In young healthy people, the more SWS that a period of sleep contains, the greater the benefit of that sleep for the retention of declarative memories (e.g., Alger et al., 2012, Barrett and Ekstrand, 1972, Born et al., 2006, Diekelmann et al., 2012, Fowler et al., 1973, Lau et al., 2010, Plihal and Born, 1997, Plihal and Born, 1999, Yaroush et al., 1971). The disruption of slow wave activity during a post-learning nap adversely affects subsequent memory performance (Garside, Arizpe, Lau, Goh, & Walsh, 2015), while enhancement of the endogenous slow oscillation using electrical (Marshall, Helgadottir, Molle, & Born, 2006) or auditory (Ngo, Martinetz, Born, & Molle, 2013) stimulation improves memory for information learnt prior to sleep. Slow oscillations are thought to trigger the reactivation of recently-acquired, memory-related activity patterns in the hippocampus (Born et al., 2006, Marshall and Born, 2007) which strengthens the memories (e.g., Diekelmann et al., 2011, Rasch et al., 2007, Rudoy et al., 2009) and, when interleaved with reactivations of related older memories, helps integrate them with pre-existing knowledge (McClelland, McNaughton, & O'Reilly, 1995). Techniques designed to induce memory reactivation using sensory cues have demonstrated a benefit for memory if the cues are provided during SWS, but not if provided during rapid eye-movement (REM) sleep or active wakefulness (e.g., Diekelmann et al., 2011, Rasch et al., 2007, Rudoy et al., 2009). However, it should be noted that there is evidence to suggest that reactivation-based declarative memory consolidation does not happen exclusively during SWS; it also occurs during quiet rest (e.g., Axmacher et al., 2008, Deuker et al., 2013, Ego-Stengel and Wilson, 2010, Groen et al., 2011, Tambini and Davachi, 2013).

Sleep spindles are associated with synaptic plasticity (Rosanova & Ulrich, 2005). Their incidence increases following learning (e.g., Gais, Molle, Helms, & Born, 2002) and has been found to correlate with overnight memory retention in some cases (Schabus et al., 2004). According to an influential model from Born and colleagues (Born et al., 2006, Marshall and Born, 2007), these sleep spindles are an important component of SWS-associated memory consolidation; these researchers propose that slow oscillations trigger spindles from the thalamus in tandem with reactivations in the hippocampus, and that it is these sleep spindles that allow the reactivations to induce plastic changes in the neocortex.

An alternative account for the benefit of SWS for declarative memory retention is that it downscales synaptic weights, improving memory by increasing the signal-to-noise ratio. That is, mechanisms reflected by slow waves may reduce the strength of synapses representing noise to such a level that they no longer interfere with memory retrieval (Tononi and Cirelli, 2003, Tononi and Cirelli, 2006).

### 1.3 Sleep and ALF

It has frequently been suggested that memory in people with ALF may not benefit from sleep in the same way that it does in healthy people (e.g., Butler et al., 2009, Holmes and Lenck-Santini, 2006, Jansari et al., 2010, Muhlert et al., 2011, Sud et al., 2014, Tramoni et al., 2011, Urbain et al., 2011, Zeman et al., 2013). A recent study of ours, however, demonstrated that sleep can benefit memory retention in people with ALF just as much as it does in healthy control participants (Atherton, Nobre, Zeman, & Butler, 2014). Nevertheless, this benefit may be largely due to sleep's passive role in protecting against interference. It remains possible that SWS-specific *active* processes of sleep-related memory consolidation are disrupted in people with ALF.

There is some evidence to suggest that epileptic activity following learning is associated with poor memory retention in people with epilepsy (Fitzgerald et al., 2013, Jokeit et al., 2001, Mameniskiene et al., 2006, Ricci et al., 2015b, Ricci et al., 2015a, Wilkinson et al., 2012). Interictal and ictal epileptiform discharges are enhanced during SWS, compared to wakefulness and REM sleep (Bazil, 2000, Bazil and Walczak, 1997, Goncharova et al., 2009, Kotagal, 2001, Mayanagi, 1977, Nazer and Dickson, 2009, Romcy-Pereira et al., 2009, Rossi et al., 1984, Sammaritano et al., 1991), and could potentially interfere with the SWS-associated brain activity patterns that facilitate memory retention in healthy people (Galer et al., 2015, Shatskikh et al., 2006, Tassinari et al., 2009, Urbain et al., 2011, Verrotti et al., 2014).

In people with transient epileptic amnesia, half of whom complain of ALF (Butler et al., 2007), the attacks of transient amnesia characteristic of the syndrome often occur upon waking from sleep, suggesting a link between seizure activity and sleep (Butler et al., 2007). Furthermore, electroencephalography in people with TEA is more likely to reveal epileptic abnormalities if performed while the person is asleep than whilst they are awake and alert (Butler et al., 2007, Zeman et al., 1998).

### 1.4 Hypothesis

The objective of the current study was to investigate the role played by SWS in the benefit of sleep for memory retention seen in patients with TEA-associated ALF. We hypothesized that people with ALF would not benefit from SWS in the same way as healthy people. In our results, this would manifest as a significant difference between the groups in terms of the relationship between the amount of SWS and the benefit of sleep for memory.

If this were found to be the case, then further investigation of this effect may provide insight into the mechanism by which SWS benefits memory in healthy people. As a first step in this direction, we investigated spindle activity; a significant group difference in SWS-associated spindle activity or its relationship with the benefit of sleep for memory retention would be more consistent with Born's model of SWS-dependent declarative memory consolidation than Tononi and Cirelli's downscaling model.

## 2 Methods

The participants and behavioural task have been fully described elsewhere (Atherton et al., 2014). They are summarised here and in the Online Supplementary material.

### 2.1 Participants

Clinical data from the eleven patients are presented in Table S1. The patients met widely-used diagnostic criteria for transient epileptic amnesia (Zeman & Butler, 2010) and complained of anterograde memory problems to their clinician indicative of ALF, describing a fading of recently acquired memories over hours-weeks. Such complaints of ALF have previously been shown to correlate with objective measures of memory retention over extended delays (Butler et al., 2009). All patients were on anticonvulsant monotherapy and, in all cases but one, had been free of seizures for at least six months prior to testing. No patients reported seizures during the experiment. The patients were matched to twelve control participants in terms of age, IQ and performance on a range of standard neuropsychological tests (see Table S2).

### 2.2 Behavioural data collection and analysis

**Fig. S1:**
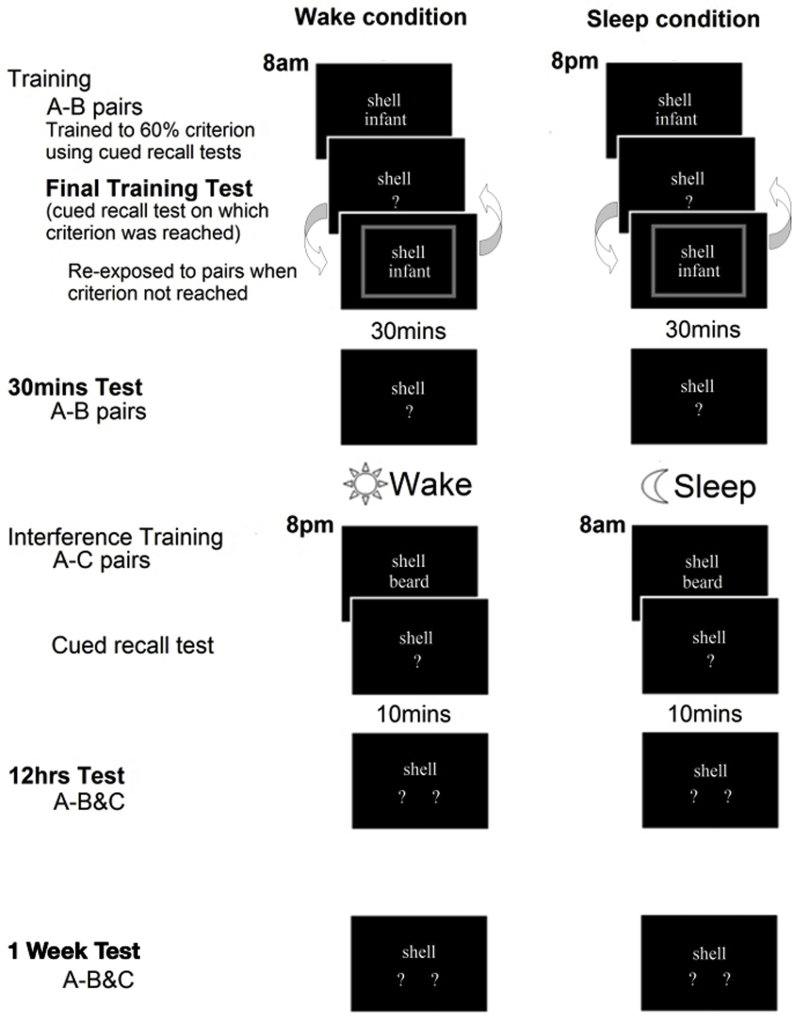
An illustration of the word-pair associates paradigm, adapted from Atherton et al. (2014). Memory performance was assessed using cued recall tests without feedback. During the initial training stage, until the participants reached criterion in one of the cued recall tests, they were repeatedly re-exposed to the full set of pairs, with coloured borders around each pair (green for correct and red for incorrect) providing feedback on their most recent response.

The behavioural paradigm is illustrated in Fig. S1. In brief, the participants were trained to 60% criterion on 30 unrelated word-pairs (A–B) either at 8 pm or at 8 am. Cued recall of the word pairs was probed after 30 min, and then again after twelve hours, following a night of sleep or a day of wakefulness. Just prior to the twelve-hour probe, participants learnt, over a single trial, an interference word-pair list (A–C) to enhance detectability of the sleep-consolidation effect (Ellenbogen, Hulbert, Stickgold, Dinges, & Thompson-Schill, 2006). All participants underwent both sleep and wake conditions, in a counterbalanced order, with 24 h in between.

There was an additional memory test one week later, performed over the telephone. It would have been preferable to do in-person testing, if practical, but telephone testing at delayed time-points has been shown to provide equivalent results (Geffen, Geffen, Bishop, & Manning, 1997) and has been employed in previous published studies of ALF (Miller, Flanagan, Mothakunnel, Mohamed, & Thayer, 2015).

Behavioural data were analysed using mixed effects ANOVAs and Bonferroni corrected post hoc pairwise comparisons.

### 2.3 Sleep data collection and analysis

To give the participants an opportunity to acclimatise to the sleep laboratory and the polysomnography (PSG) equipment, they spent an adaptation night in the sleep laboratory the night before the experiment began.

A Somnoscreen PSG system (SOMNOmedics, Randersacker, Germany) and Domino software (SOMNOmedics, Randersacker, Germany) were used to collect PSG data. The following recording electrodes from the 10–20 system were used: O1; O2; T5; P3; P4; T6; T3; C3; C4; T4; F7; F3; F4; F8; FP1; FP2. Cz was used as the online reference. In addition, the electrooculogram, chin electromyogram and electrocardiogram were recorded. The sleep electroencephalography (EEG) signals were sampled at 256 Hz and filtered using a .2–128 Hz bandpass. Recording electrodes were re-referenced to the contralateral mastoid. Sleep stages were scored for each 30 sec of data by an experienced rater (A.S.L.) according to the Rechtschaffen and Kales (1968) criteria. The scorer was blind as to whether each participant belonged to the patient or the control group.

Sleep latency was calculated as the time from lights off until sleep onset. Sleep efficiency was calculated as the total sleep time as a percentage of the time in bed. As with the other sleep stages, the percentage of SWS was calculated as the number of minutes spent in SWS expressed as a percentage of the total sleep time. The benefit of sleep for memory retention in each individual was calculated as the number of A–B pairs forgotten between the 30-min and the delayed (12-h or 1-week) tests in the wake condition minus that in the sleep condition; therefore, the more positive the value, the greater the benefit of sleep for memory retention. Relationships between the percentage of SWS and the benefit of sleep for memory retention were probed using two-tailed Pearson correlation tests. To minimize multiple comparisons problems, we restricted our correlational analyses to the sleep stage of a priori interest: SWS. However, data from the other sleep stages can be found in the Supplementary material.

The patient neural data from the sleep-condition night were examined for epileptiform abnormalities by a clinical neurophysiologist (V.D.).

#### 2.3.1 Spindle analyses

The spindle detection procedure is a modified variant of the one described in O'Reilly and Nielsen (2014) with parameters adjusted such that it performed optimally when validated against spindle sets identified visually by an expert (A.S.L.) (For details see Supplementary methods).

Spindle incidence in SWS was calculated as the total number of spindles detected during SWS divided by the number of artefact-free SWS minutes. This calculation was performed for each channel, and these values were averaged to produce a single value for each participant. It should be noted that the first control participant had a different montage (Fpz, Fz, C3, C4, Pz, and Oz only).

Relationships between the incidence of spindles during SWS and the benefit of sleep for memory retention were probed using two-tailed Pearson correlation tests. To minimize multiple comparisons problems, we restricted our correlational analyses to the sleep stage of a priori interest: SWS. However, data from NREM2, in which sleep spindles are prevalent, can be found in the Supplementary material.

## 3 Results

### 3.1 Memory performance

The behavioural data from the current study, with the exception of those from the follow-up test after one week, have been published previously (Atherton et al., 2014) and are summarised in Table S3. While there were no significant group differences in learning, the patients showed ALF for the word-pairs over twelve hours. The participants demonstrated a benefit of sleep for memory retention over twelve hours, and this benefit was no smaller in magnitude for the patients than the controls.

When the one-week data (see Fig. S2b) were incorporated into the main analysis – a mixed-effects ANOVA with A–B pair performance as the dependent variable, sleep condition (two levels: night of sleep and day of wakefulness following learning) and retrieval time point (four levels: final training test, 30-min test, 12-h test and one-week test) as the within-subjects factors and group as the between-subjects factor – the significance of the main effects and interaction effects remained the same, except that the main effect of group now reached significance, with patients performing significantly more poorly than controls overall [F_(1,21)_ = 5.14, *p* = .034, estimated marginal means (EMMs) and standard errors of the mean (SEMs): 17.38 ± .72 and 15.02 ± .75].

**Fig. 1:**
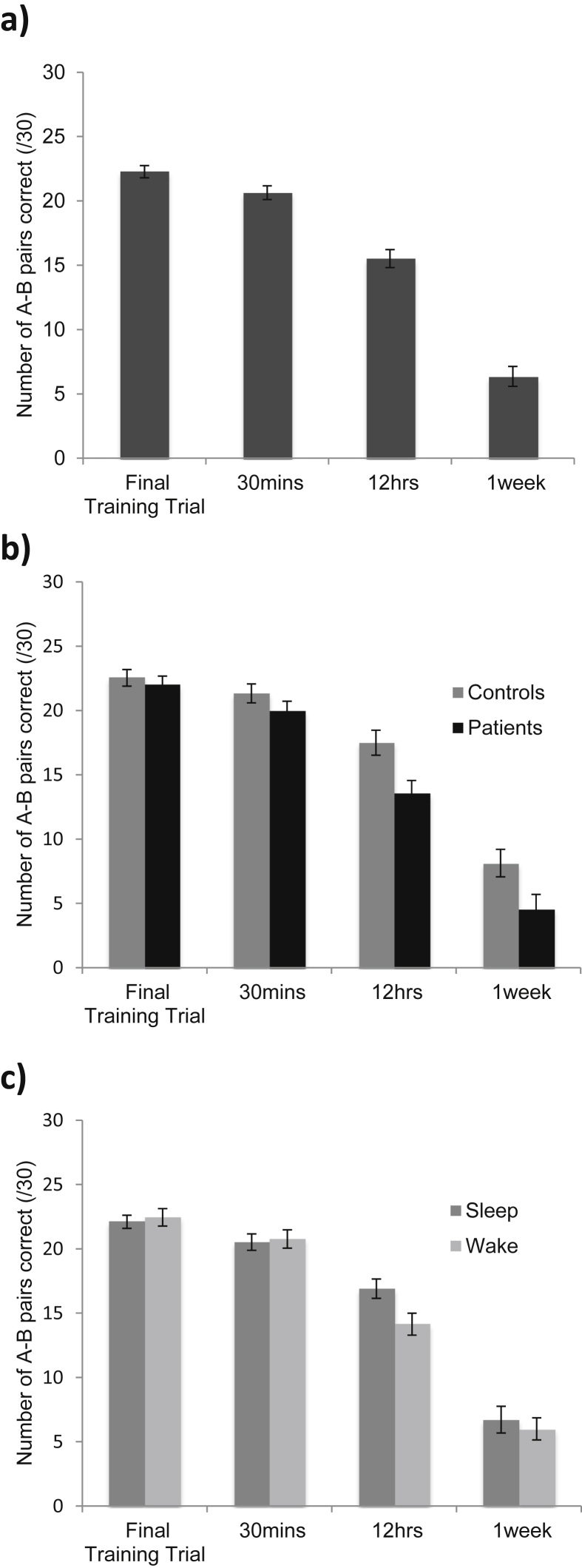
Estimated marginal means and standard errors of the mean for the behavioural analysis.

Performance declined across the retrieval time points: there was a main effect of retrieval time point [F_(1.61, 33.84)_ = 303.08, *p* < .001], with participants performing progressively more poorly on each test (see Fig. 1a for the EMMs and SEMs, *p* < .001 for every pairwise comparison). The patients performed significantly more poorly than the controls on the later retrieval time points, but not the early ones, consistent with the typical profile of ALF: there was an interaction between retrieval time point and group [F_(1.61, 33.84)_ = 4.03, *p* = .035], and pairwise comparisons revealed a significant group difference on the 12-h test (*p* = .01) and the one-week test (*p* = .032) but not on the final training test (*p* = .57) or the 30-min test (*p* = .21). See Fig. 1b for the EMMs and SEMs. There was a benefit of sleep for memory retention over 12 h: there was an interaction between sleep condition and retrieval time point [F_(1.64, 34.46)_ = 5.76, *p* = .01], with participants performing significantly better in the sleep condition than the wake condition on the 12-h test (*p* = .002) but not on the final training test (*p* = .64) or the 30-min test (*p* = .78). However, there was no significant difference between the night of sleep and day of wakefulness conditions on the one-week test (*p* = .53). See Fig. 1c for the EMMs and SEMs. The benefit of sleep for memory retention was no smaller in magnitude for the patients than the controls: there was no significant interaction between sleep condition, retrieval time point and group [F_(1.64, 34.46)_ = .43, *p* = .62].

### 3.2 Sleep

Independent samples *t*-tests revealed that, during the night of the sleep condition, the patients and controls did not significantly differ in terms of total sleep time (406.18 ± 15.92 min in patients and 376.21 ± 15.43 min in controls, *p* = .19), sleep latency (11.96 ± 3.36 and 13.54 ± 5.36 min, *p* = .81), sleep efficiency (81.38 ± 2.85% and 81.13 ± 2.52%, *p* = .95), number of awakenings (38.27 ± 3.31 and 35.33 ± 4.00, *p* = .58), or minutes spent awake between sleep onset and final awakening (78.32 ± 13.39 and 64.00 ± 11.54, *p* = .43). They also did not differ in terms of percentage of total sleep time spent in each sleep stage (see Table 1). A total of five minutes of data across all participants could not be identified as a particular sleep stage or the waking state: 1.5 min in the controls and 3.5 min in the patients.

Independent samples *t*-tests revealed no significant difference between patients and controls in sleep spindle incidence during SWS (3.57 ± .38 and 3.06 ± .49 spindles per minute, *p* = .43).

No epileptiform abnormalities were seen in any of the patients' sleep-condition EEGs.

#### 3.2.1 Association between memory performance and sleep

**Fig. 2:**
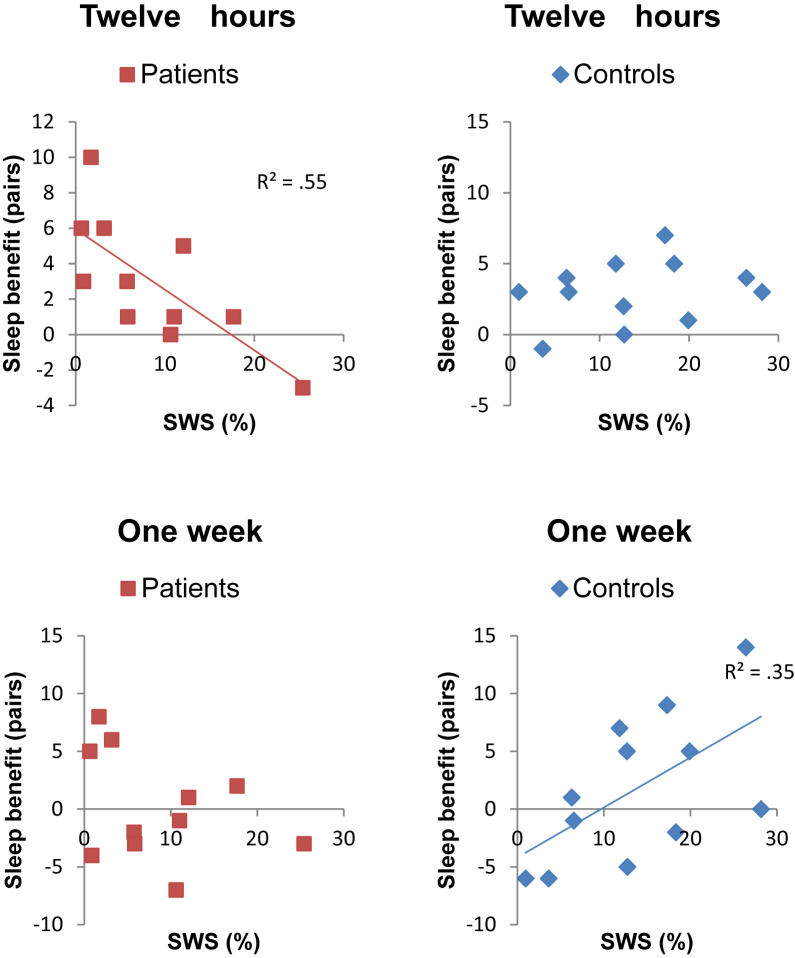
The benefit of post-learning sleep for memory retention over twelve hours (top charts) and one week (bottom charts) plotted against the percentage of total sleep time spent in SWS during the sleep condition night. The lines of best fit and R^2^ values are displayed for the significant correlations. The patients showed a significant negative correlation between SWS (%) and the benefit of post-learning sleep for memory retention over twelve hours. This correlation was not seen in the controls and the correlation significantly differed between the two groups. The controls showed a significant positive correlation between SWS (%) and the benefit of post-learning sleep for memory retention over one week. This correlation was not seen in the patients and the correlation significantly differed between the two groups.

In a two-tailed Pearson correlation test, the patients showed a significant negative correlation between the percentage of SWS and the benefit of sleep for memory retention over twelve hours (*r* = −.741, *p* = .009, see Fig. 2). The controls did not show this negative correlation; their Pearson correlation coefficient was positive and non-significant (*r* = .266, *p* = .403). A Fisher's transformation analysis revealed that the correlation was significantly different in the two groups (*z* = 2.52, *p* = .012).

The controls showed a significant positive correlation between the percentage of SWS and the benefit of post-learning sleep for memory retention over one week (*r* = .589, *p* = .044, see Fig. 2). The patients did not show this positive correlation. The patients' Pearson correlation coefficient was negative and non-significant (*r* = −.354, *p* = .29). The Fisher's transformation analysis revealed that the correlation was significantly different in the two groups (*z* = 2.15, *p* = .032).

When the three controls and the two patients who had been excluded (for group matching and outlier behaviour reasons, as described in the Supplementary material) were included in these correlational analyses, the pattern of the results remained the same (as detailed in the Supplementary material).

Two-tailed Pearson correlation tests revealed no significant relationship between spindle incidence in SWS and the benefit of sleep for memory retention in the controls, nor in the patients, and no significant group difference (see Fig. S4, twelve hours: *r* = −.062, *p* = .85 and *r* = .507, *p* = .11, *z* = −1.28, *p* = .20; one week: *r* = .014, *p* = .97 and *r* = .40, *p* = .22, *z* = −.85, *p* = .39).

## 4 Discussion

This study revealed a striking difference in the relationship between SWS and the benefit of sleep for memory retention in patients with TEA-associated ALF and healthy older adults, while objective sleep quality and architecture did not differ between the groups. The amount of SWS was positively correlated with the benefit of post-learning sleep for memory retention over one week in healthy older adults. However, this relationship was not found in the patients. Furthermore, the amount of SWS was negatively correlated with the benefit of sleep for memory retention over twelve hours in patients. This relationship was not observed in the controls. Indeed, these correlations were significantly different in the two groups. These opposing results suggest that the role played by sleep in memory retention is not the same in the two groups, and that SWS-associated brain activity after learning is deleterious, rather than advantageous, for memory retention in people with TEA-associated ALF. We next consider potential reasons why this might be so.

SWS may be deleterious to memory in these patients because it promotes epileptic activity. Epileptic activity could disrupt the synaptic weights that constitute a memory trace; the conditions for the induction of synaptic plasticity are satisfied during interictal epileptic spike activity (Buzsaki, 1989). Epileptic activity might scramble activity patterns during reactivations, or disrupt post-reactivation reconsolidation. There was no evidence of epileptic activity in the patients' EEGs in this study, but standard surface EEG electrodes have limited sensitivity to medial temporal lobe (MTL) epileptiform activity (e.g., Javidan, 2012). Future studies should use more sensitive methods, such as anterior temporal, mandibular notch, sphenoidal and/or nasopharyngeal electrodes.

Alternatively, SWS may interact abnormally with memory traces in TEA-associated ALF even in the absence of epileptic spike activity. For example, it is possible that the mechanisms that normally protect important memory traces from sleep's synaptic downscaling process (Hardt, Nader, & Nadel, 2013) are impaired in the patients. Another possibility is that the positive effects of reactivation-mediated consolidation are disrupted. The normal temporal synchronisation between hippocampal activity, spindles and/or neocortical slow waves could be disturbed (Clemens et al., 2007, Holler and Trinka, 2015). Neocortical slow waves might also trigger chaotic hippocampal activity (Holler & Trinka, 2015), which could even reflect the pattern of prior epileptiform activity (Bower et al., 2015), rather than faithful memory reactivations. Finally, it may be that the SWS-associated brain activity patterns are entirely normal but that the memory reactivations exert a negative, rather than positive, effect on memory in this patient group. During SWS in healthy people, new memories are thought to be reactivated in an interleaved manner with related older memories, leading them to integrate. Unfortunately, if memories are abnormal in people with TEA-associated ALF as a result of a poorly functioning hippocampal complex, these patients might have difficulty separating memories with overlapping components (Bartko et al., 2010, Bussey et al., 2005, Cowell et al., 2006, Graham et al., 2010, Hardt et al., 2013, Lee et al., 2012, Yassa and Stark, 2011), and the reactivation of related memories might promote catastrophic interference. However, if the patients were suffering from a problem related to reactivation-mediated consolidation as described by Born and colleagues (Born et al., 2006, Marshall and Born, 2007), one might expect a group difference in the relationship between SWS spindle incidence and the benefit of sleep for memory, and this was not found.

Future research should investigate the means by which SWS adversely affects memory in patients with ALF in order to provide further insight into the neural basis of ALF and the mechanism by which SWS facilitates memory in healthy people.

The current study provides only correlational evidence of a negative relationship between SWS and the benefit of sleep for memory retention in people with TEA-associated ALF. To investigate causality, slow waves could be manipulated in these patients. Slow waves can be enhanced or suppressed using acoustic stimuli. It is possible to interrupt slow wave activity by presenting acoustic stimuli whenever slow waves are detected in the EEG (e.g., Landsness et al., 2009). Alternatively, if the sounds are presented in a rhythmic fashion at the appropriate frequency, in phase with the ongoing slow oscillations, they can be used to enhance slow oscillation activity through entrainment (e.g., Ngo et al., 2013). If the mechanisms reflected by slow waves are indeed deleterious for the memories of people with TEA-associated ALF, slow wave suppression should enhance memory retention and slow wave enhancement should exacerbate forgetting.

The results from the healthy control group are interesting in themselves. To date, very little research has been done on the role of SWS in declarative memory retention in healthy older adults. Some studies suggest that, while SWS is reduced in older people, the relationship between SWS and memory retention is preserved (e.g., Backhaus et al., 2007, Mander et al., 2013). However, other studies have failed to find a relationship between SWS and memory retention in older adults (e.g., Scullin, 2013). Our study replicated the positive relationship between amount of SWS and memory that has been identified previously in young healthy adults. Interestingly, this was only detected in the one-week measure, and not at twelve hours after learning. Furthermore, the group as a whole performed significantly better in the sleep condition than the wakefulness condition on the twelve-hours test, but not on the one-week test. This pattern of results implies that (a) a night of sleep was beneficial for memory compared to a day of wakefulness irrespective of the amount of SWS that it contained, perhaps because a large part of the benefit at this stage was mediated by protection from retroactive interference, and (b) sleep immediately after learning only had a long-term benefit for memory retention if that sleep contained a high level of SWS (and therefore was presumably actively consolidating the memories). However, it should be noted that it is possible that our study was simply not sufficiently sensitive to detect a benefit of sleep for memory retention over one week and/or a correlation between SWS and sleep benefit over twelve hours; even with the originally excluded participants included in the correlational analyses, the sample size was relatively small, with only fourteen control participants.

It should also be noted that we did not detect a relationship between spindle incidence and the benefit of sleep for memory retention. Relatively few studies have investigated this relationship in older adults (e.g., Rauchs et al., 2008, Seeck-Hirschner et al., 2012) and it may be that the role of spindles in memory consolidation diminishes with age.

It does not appear that any part of the benefit of sleep for memory retention in people with TEA-associated ALF is mediated by SWS. Nevertheless, sleep must have a positive role in declarative memory retention in these patients as they showed a behavioural benefit. As we have discussed, sleep may make a major contribution to declarative memory retention simply by minimizing interference. This role would be especially important if the patients' memory traces or consolidation processes were particularly vulnerable to interference. While it is not yet known if people with TEA-associated ALF are especially vulnerable to interference, there is ample evidence that MTL pathology results in increased susceptibility to interference (e.g., Bartko et al., 2010, Cowan et al., 2004, Cowell et al., 2006, Dewar et al., 2009, Warrington and Weiskrantz, 1978). According to many theories of MTL function, the hippocampus is critical to the brain's ability to represent memories in such a way that they are distinguishable from each other (e.g., Cowell et al., 2010, Graham et al., 2010; Marr, 1971, O'Reilly and McClelland, 1994, Treves and Rolls, 1994, Yassa and Stark, 2011). If the function of this structure was compromised, memories with overlapping neocortical components would be more likely to interfere with one another. Future work should involve assessing whether people with TEA-associated ALF are more susceptible to interference than healthy people.

However, it remains possible that a different stage of sleep has taken over the memory-enhancing role of SWS in this patient population. The relationship between other sleep stages and the benefit of sleep for memory should be investigated in a future study with a larger sample size. Due to the limited sample size in the current study, the need for multiple comparisons corrections would have been problematic if we were to perform exploratory correlational analyses, investigating sleep stages that were not of a priori interest; for this reason, these data are presented only in the Supplementary material. The positive correlation in Fig. S3 in the patient group between the percentage of NREM2 and the benefit of sleep for memory retention over twelve hours raises the possibility that NREM2 might be particularly advantageous for memory retention in these patients, but one might also expect this result if patients are simply benefiting from minimized interference; in REM sleep, brain activity is relatively similar to that in the waking state (e.g., Born et al., 2006, Hasselmo, 1999, Rechtschaffen and Kales, 1968) and therefore might be interfering rather than protective, leaving NREM2 as the only safe haven for memory traces in a patient group in which SWS is disruptive.

It should be noted that our results cannot be generalised to all kinds of declarative memory, since our task used only verbal material. Future studies should use a range of different sleep-sensitive tasks. Furthermore, as cortisol levels peak between eight and nine in the morning (Debono et al., 2009), and cortisol is known to influence both memory performance in older adults (Hidalgo, Almela, Pulopulos, & Salvador, 2016) and epileptic activity (van Campen et al., 2015, van Campen et al., 2016), future studies should use testing time-points that fall outside the cortisol response peak. It might also be interesting to measure cortisol levels in order to investigate any potential group differences.

In conclusion, while people with TEA-associated ALF have a normal behavioural benefit of sleep for memory retention, the more SWS they have on the post-learning night, the smaller the benefit of the sleep for their memory. This apparently deleterious effect of SWS may be due to promotion of epileptic activity, although none was detected in this study, or to an aberrant interaction between memory traces and SWS-associated brain activity. Future studies should investigate the mechanism by which SWS has this apparently negative impact. This is of theoretical interest because it contributes to our knowledge of the process of memory consolidation. However, understanding the mechanisms of ALF is also of clinical importance because it may help in the development of treatments. While ALF is particularly common, and is seen in an especially pure form, in TEA, it is also observed much more widely in epilepsy and has a considerable impact on quality of life.

## Figures and Tables

**Table 1:** Time spent in each sleep stage. The means (and standard errors of the mean) are presented for the percentage of total sleep time spent in each sleep stage by the patients and the controls. Independent samples *t*-tests found no significant group differences in time spent in each sleep stage.

	Patients	Controls	*p* value
NREM1 (%)	19.90 (±1.81)	16.35 (±1.92)	.19
NREM2 (%)	51.43 (±2.15)	48.91 (±2.38)	.45
SWS (%)	8.62 (±2.34)	13.73 (±2.49)	.15
REM (%)	20.04 (±.80)	21.01 (±1.18)	.52

## Appendix A Supplementary data

Fig. S2a A–B pair performance in the final training test, the 30-min test and the 12-h test in the sleep and wake conditions of the word-pair associates task, in people with TEA-associated ALF and control participants, taken from Atherton et al. (2014). Error bars represent standard errors of the mean. Fig. S2a

Fig. S2b A–B pair performance in the 1-week test in the sleep and wake conditions of the word-pair associates task, in people with TEA-associated ALF and control participants. Error bars represent standard errors of the mean. Fig. S2b

Fig. S3 The benefit of post-learning sleep for memory retention over twelve hours (top charts) and one week (bottom charts) plotted against the percentage of total sleep time spent in REM sleep (left charts) and NREM2 sleep (right charts) during the sleep condition night. The lines of best fit and R^2^ values are displayed for the significant correlations. The patients showed a significant positive correlation between NREM2 (%) and the benefit of post-learning sleep for memory retention over twelve hours. This correlation was not seen in the controls and the correlation significantly differed between the two groups. Fig. S3

Fig. S4 The benefit of post-learning sleep for memory retention over twelve hours (top charts) and one week (bottom charts) plotted against the spindle incidence in SWS (the number of spindles in SWS/the total number of artefact free SWS min) during the sleep condition night. There were no significant results. Fig. S4

Fig. S5 The benefit of post-learning sleep for memory retention over twelve hours (top charts) and one week (bottom charts) plotted against the spindle incidence in NREM2 (the number of spindles in NREM2/the total number of artefact free NREM2 min) during the sleep condition night. There were no significant results. Fig. S5

Table S1 TEA patient information, adapted from Atherton et al. (2014). AEDs = Anti-Epileptic Drugs that the patients were taking when they took part in the sleep experiment, LEV = Levetiracetam, CBZ = Carbamazepine, LTG = Lamotrigine, SVP = Sodium Valproate. Table S1

Table S2 Participant information, adapted from Atherton et al. (2014). Means with SEMs in brackets. Patients and controls did not significantly differ in terms of age, years of full-time education or test scores (ps > .05). ^a^NART (Nelson, 1982; Nelson & Willison, 1991); ^b^WAIS = Wechsler Abbreviated Intelligence Scale (Wechsler, 1955); ^c^WASI = Wechsler Abbreviated Scale of Intelligence (Wechsler, 1999); ^d^WMS-III = Wechsler Memory Scale-III (Wechsler, 1997); ^e^Rey-Osterrieth complex figure (Rey, 1941); ^f^RMT (Warrington, 1984); ^g^GNT (McKenna & Warrington, 1980); ^h^DKEFS = Delis-Kaplan Executive Function System (Delis et al., 2001); ^i^HADS (Zigmond & Snaith, 1983). Table S2

Table S3 Performance on the word-pair associates task, adapted from Atherton et al. (2014). Means with SEMs in brackets. The three A–B word-pair tests that are plotted in Fig. 2a are in boldface. Table S3

## References

[bib1] Alger S.E., Lau H., Fishbein W. (2012). Slow wave sleep during a daytime nap is necessary for protection from subsequent interference and long-term retention. Neurobiology of Learning and Memory.

[bib2] Atherton K.E., Nobre A.C., Zeman A.Z., Butler C.R. (2014). Sleep-dependent memory consolidation and accelerated forgetting. Cortex.

[bib3] Axmacher N., Elger C.E., Fell J. (2008). Ripples in the medial temporal lobe are relevant for human memory consolidation. Brain.

[bib4] Backhaus J., Born J., Hoeckesfeld R., Fokuhl S., Hohagen F., Junghanns K. (2007). Midlife decline in declarative memory consolidation is correlated with a decline in slow wave sleep. Learning & Memory.

[bib5] Barrett T.R., Ekstrand B.R. (1972). Effect of sleep on memory. 3. Controlling for time-of-day effects. Journal of Experimental Psychology.

[bib6] Bartko S.J., Cowell R.A., Winters B.D., Bussey T.J., Saksida L.M. (2010). Heightened susceptibility to interference in an animal model of amnesia: Impairment in encoding, storage, retrieval–or all three?. Neuropsychologia.

[bib7] Bazil C.W. (2000). Sleep and epilepsy. Current Opinion in Neurology.

[bib8] Bazil C.W., Walczak T.S. (1997). Effects of sleep and sleep stage on epileptic and nonepileptic seizures. Epilepsia.

[bib9] Bell B.D., Giovagnoli A.R. (2007). Recent innovative studies of memory in temporal lobe epilepsy. Neuropsychology Review.

[bib10] Born J., Rasch B., Gais S. (2006). Sleep to remember. The Neuroscientist.

[bib11] Bower M.R., Stead M., Bower R.S., Kucewicz M.T., Sulc V., Cimbalnik J. (2015). Evidence for consolidation of neuronal assemblies after seizures in humans. The Journal of Neuroscience.

[bib12] Brown G.D., Neath I., Chater N. (2007). A temporal ratio model of memory. Psychological Review.

[bib13] Bussey T.J., Saksida L.M., Murray E.A. (2005). The perceptual-mnemonic/feature conjunction model of perirhinal cortex function. The Quarterly Journal of Experimental Psychology B.

[bib14] Butler C.R., Bhaduri A., Acosta-Cabronero J., Nestor P.J., Kapur N., Graham K.S. (2009). Transient epileptic amnesia: Regional brain atrophy and its relationship to memory deficits. Brain.

[bib15] Butler C.R., Graham K.S., Hodges J.R., Kapur N., Wardlaw J.M., Zeman A.Z. (2007). The syndrome of transient epileptic amnesia. Annals of Neurology.

[bib16] Butler C.R., Zeman A.Z. (2008). Recent insights into the impairment of memory in epilepsy: Transient epileptic amnesia, accelerated long-term forgetting and remote memory impairment. Brain.

[bib17] Buzsaki G. (1989). Two-stage model of memory trace formation: A role for “noisy” brain states. Neuroscience.

[bib18] van Campen J.S., Hompe E.L., Jansen F.E., Velis D.N., Otte W.M., van de Berg F. (2016). Cortisol fluctuations relate to interictal epileptiform discharges in stress sensitive epilepsy. Brain.

[bib19] van Campen J.S., Valentijn F.A., Jansen F.E., Joels M., Braun K.P. (2015). Seizure occurrence and the circadian rhythm of cortisol: A systematic review. Epilepsy & Behavior.

[bib20] Clemens Z., Molle M., Eross L., Barsi P., Halasz P., Born J. (2007). Temporal coupling of parahippocampal ripples, sleep spindles and slow oscillations in humans. Brain.

[bib21] Cowan N., Beschin N., Della Sala S. (2004). Verbal recall in amnesiacs under conditions of diminished retroactive interference. Brain.

[bib22] Cowell R.A., Bussey T.J., Saksida L.M. (2006). Why does brain damage impair memory? A connectionist model of object recognition memory in perirhinal cortex. The Journal of Neuroscience.

[bib23] Cowell R.A., Bussey T.J., Saksida L.M. (2010). Components of recognition memory: Dissociable cognitive processes or just differences in representational complexity?. Hippocampus.

[bib24] Debono M., Ghobadi C., Rostami-Hodjegan A., Huatan H., Campbell M.J., Newell-Price J. (2009). Modified-release hydrocortisone to provide circadian cortisol profiles. The Journal of Clinical Endocrinology and Metabolism.

[bib25] Deuker L., Olligs J., Fell J., Kranz T.A., Mormann F., Montag C. (2013). Memory consolidation by replay of stimulus-specific neural activity. The Journal of Neuroscience.

[bib26] Dewar M., Garcia Y.F., Cowan N., Della Sala S. (2009). Delaying interference enhances memory consolidation in amnesic patients. Neuropsychology.

[bib27] Diekelmann S., Biggel S., Rasch B., Born J. (2012). Offline consolidation of memory varies with time in slow wave sleep and can be accelerated by cuing memory reactivations. Neurobiology of Learning and Memory.

[bib28] Diekelmann S., Buchel C., Born J., Rasch B. (2011). Labile or stable: Opposing consequences for memory when reactivated during waking and sleep. Nature Neuroscience.

[bib29] Ego-Stengel V., Wilson M.A. (2010). Disruption of ripple-associated hippocampal activity during rest impairs spatial learning in the rat. Hippocampus.

[bib30] Ellenbogen J.M., Hulbert J.C., Stickgold R., Dinges D.F., Thompson-Schill S.L. (2006). Interfering with theories of sleep and memory: Sleep, declarative memory, and associative interference. Current Biology.

[bib31] Fitzgerald Z., Thayer Z., Mohamed A., Miller L.A. (2013). Examining factors related to accelerated long-term forgetting in epilepsy using ambulatory EEG monitoring. Epilepsia.

[bib32] Fowler M.J., Sullivan M.J., Ekstrand B.R. (1973). Sleep and memory. Science.

[bib33] Gais S., Molle M., Helms K., Born J. (2002). Learning-dependent increases in sleep spindle density. The Journal of Neuroscience.

[bib34] Galer S., Urbain C., De Tiège X., Emeriau M., Leproult R., Deliens G. (2015). Impaired sleep-related consolidation of declarative memories in idiopathic focal epilepsies of childhood. Epilepsy & Behavior.

[bib35] Garside P., Arizpe J., Lau C.I., Goh C., Walsh V. (2015). Cross-hemispheric alternating current stimulation during a nap disrupts slow wave activity and associated memory consolidation. Brain Stimulation.

[bib36] Geffen G.M., Geffen L., Bishop K., Manning L. (1997). Extended delayed recall of avlt word lists: Effects of age and sex on adult performance. Australian Journal of Psychology.

[bib37] Goncharova, Zaveri H.P., Duckrow R.B., Novotny E.J., Spencer S.S. (2009). Spatial distribution of intracranially recorded spikes in medial and lateral temporal epilepsies. Epilepsia.

[bib38] Graham K.S., Barense M.D., Lee A.C. (2010). Going beyond LTM in the MTL: A synthesis of neuropsychological and neuroimaging findings on the role of the medial temporal lobe in memory and perception. Neuropsychologia.

[bib39] Groen G., Sokolov A.N., Jonas C., Roebling R., Spitzer M. (2011). Increased resting-state perfusion after repeated encoding is related to later retrieval of declarative associative memories. PLoS One.

[bib40] Hardt O., Nader K., Nadel L. (2013). Decay happens: The role of active forgetting in memory. Trends in Cognitive Sciences.

[bib41] Hasselmo M.E. (1999). Neuromodulation: Acetylcholine and memory consolidation. Trends in Cognitive Sciences.

[bib42] Hidalgo V., Almela M., Pulopulos M.M., Salvador A. (2016). Memory performance is related to the cortisol awakening response in older people, but not to the diurnal cortisol slope. Psychoneuroendocrinology.

[bib43] Holler Y., Trinka E. (2015). Is there a relation between EEG-slow waves and memory dysfunction in epilepsy? A critical appraisal. Frontiers in Human Neuroscience.

[bib44] Holmes G.L., Lenck-Santini P.P. (2006). Role of interictal epileptiform abnormalities in cognitive impairment. Epilepsy & Behavior.

[bib45] Jansari A.S., Davis K., McGibbon T., Firminger S., Kapur N. (2010). When “long-term memory” no longer means “forever”: Analysis of accelerated long-term forgetting in a patient with temporal lobe epilepsy. Neuropsychologia.

[bib46] Javidan M. (2012). Electroencephalography in mesial temporal lobe epilepsy: A review. Epilepsy Research and Treatment.

[bib47] Jokeit H., Daamen M., Zang H., Janszky J., Ebner A. (2001). Seizures accelerate forgetting in patients with left-sided temporal lobe epilepsy. Neurology.

[bib48] Kotagal P. (2001). The relationship between sleep and epilepsy. Seminars in Pediatric Neurology.

[bib49] Landsness E.C., Crupi D., Hulse B.K., Peterson M.J., Huber R., Ansari H. (2009). Sleep-dependent improvement in visuomotor learning: A causal role for slow waves. Sleep.

[bib50] Lau H., Tucker M.A., Fishbein W. (2010). Daytime napping: Effects on human direct associative and relational memory. Neurobiology of Learning and Memory.

[bib51] Lee A.C., Yeung L.K., Barense M.D. (2012). The hippocampus and visual perception. Frontiers in Human Neuroscience.

[bib52] Mameniskiene R., Jatuzis D., Kaubrys G., Budrys V. (2006). The decay of memory between delayed and long-term recall in patients with temporal lobe epilepsy. Epilepsy & Behavior.

[bib53] Mander B.A., Rao V., Lu B., Saletin J.M., Lindquist J.R., Ancoli-Israel S. (2013). Prefrontal atrophy, disrupted NREM slow waves and impaired hippocampal-dependent memory in aging. Nature Neuroscience.

[bib54] Marr D. (1971). Simple memory: A theory for archicortex. Philosophical Transactions of the Royal Society of London B Biological Sciences.

[bib55] Marshall L., Born J. (2007). The contribution of sleep to hippocampus-dependent memory consolidation. Trends in Cognitive Sciences.

[bib56] Marshall L., Helgadottir H., Molle M., Born J. (2006). Boosting slow oscillations during sleep potentiates memory. Nature.

[bib57] Mayanagi Y. (1977). The influence of natural sleep on focal spiking in experimental temporal lobe epilepsy in the monkey. Electroencephalography and Clinical Neurophysiology.

[bib58] McClelland J.L., McNaughton B.L., O'Reilly R.C. (1995). Why there are complementary learning systems in the hippocampus and neocortex: Insights from the successes and failures of connectionist models of learning and memory. Psychological Review.

[bib59] Mednick S.C., Cai D.J., Shuman T., Anagnostaras S., Wixted J.T. (2011). An opportunistic theory of cellular and systems consolidation. Trends in Neurosciences.

[bib60] Miller L.A., Flanagan E., Mothakunnel A., Mohamed A., Thayer Z. (2015). Old dogs with new tricks: Detecting accelerated long-term forgetting by extending traditional measures. Epilepsy & Behavior.

[bib61] Muhlert N., Grunewald R.A., Hunkin N.M., Reuber M., Howell S., Reynders H. (2011). Accelerated long-term forgetting in temporal lobe but not idiopathic generalised epilepsy. Neuropsychologia.

[bib62] Nazer F., Dickson C.T. (2009). Slow oscillation state facilitates epileptiform events in the hippocampus. Journal of Neurophysiology.

[bib63] Ngo H.V., Martinetz T., Born J., Molle M. (2013). Auditory closed-loop stimulation of the sleep slow oscillation enhances memory. Neuron.

[bib64] O'Reilly R.C., McClelland J.L. (1994). Hippocampal conjunctive encoding, storage, and recall: Avoiding a trade-off. Hippocampus.

[bib65] O'Reilly C., Nielsen T. (2014). Assessing EEG sleep spindle propagation. Part 2: Experimental characterization. Journal of Neuroscience Methods.

[bib66] Plihal W., Born J. (1997). Effects of early and late nocturnal sleep on declarative and procedural memory. Journal of Cognitive Neuroscience.

[bib67] Plihal W., Born J. (1999). Effects of early and late nocturnal sleep on priming and spatial memory. Psychophysiology.

[bib68] Rasch B., Buchel C., Gais S., Born J. (2007). Odor cues during slow-wave sleep prompt declarative memory consolidation. Science.

[bib69] Rauchs G., Schabus M., Parapatics S., Bertran F., Clochon P., Hot P. (2008). Is there a link between sleep changes and memory in Alzheimer's disease?. NeuroReport.

[bib70] Rechtschaffen A., Kales A. (1968). A manual of standardized terminology, techniques and scoring system for sleep stages of human subjects.

[bib71] Ricci M., Mohamed A., Savage G., Boserio J., Miller L.A. (2015). The impact of epileptiform abnormalities and hippocampal lesions on retention of recent autobiographical experiences: Adding insult to injury?. Neuropsychologia.

[bib72] Ricci M., Mohamed A., Savage G., Miller L.A. (2015). Disruption of learning and long-term retention of prose passages in patients with focal epilepsy. Epilepsy & Behavior.

[bib73] Romcy-Pereira R.N., Leite J.P., Garcia-Cairasco N. (2009). Synaptic plasticity along the sleep-wake cycle: Implications for epilepsy. Epilepsy & Behavior.

[bib74] Rosanova M., Ulrich D. (2005). Pattern-specific associative long-term potentiation induced by a sleep spindle-related spike train. The Journal of Neuroscience.

[bib75] Rossi G.F., Colicchio G., Pola P. (1984). Interictal epileptic activity during sleep: A stereo-EEG study in patients with partial epilepsy. Electroencephalography and Clinical Neurophysiology.

[bib76] Rudoy J.D., Voss J.L., Westerberg C.E., Paller K.A. (2009). Strengthening individual memories by reactivating them during sleep. Science.

[bib77] Sammaritano M., Gigli G.L., Gotman J. (1991). Interictal spiking during wakefulness and sleep and the localization of foci in temporal lobe epilepsy. Neurology.

[bib78] Schabus M., Gruber G., Parapatics S., Sauter C., Klosch G., Anderer P. (2004). Sleep spindles and their significance for declarative memory consolidation. Sleep.

[bib79] Scullin M.K. (2013). Sleep, memory, and aging: The link between slow-wave sleep and episodic memory changes from younger to older adults. Psychology and Aging.

[bib80] Seeck-Hirschner M., Baier P.C., Weinhold S.L., Dittmar M., Heiermann S., Aldenhoff J.B. (2012). Declarative memory performance is associated with the number of sleep spindles in elderly women. The American Journal of Geriatric Psychiatry.

[bib81] Shatskikh T.N., Raghavendra M., Zhao Q., Cui Z., Holmes G.L. (2006). Electrical induction of spikes in the hippocampus impairs recognition capacity and spatial memory in rats. Epilepsy & Behavior.

[bib82] Sud S., Sadaka Y., Massicotte C., Smith M.L., Bradbury L., Go C. (2014). Memory consolidation in children with epilepsy: Does sleep matter?. Epilepsy & Behavior.

[bib83] Tambini A., Davachi L. (2013). Persistence of hippocampal multivoxel patterns into postencoding rest is related to memory. Proceedings of the National Academy of Sciences of the United States of America.

[bib84] Tassinari C.A., Cantalupo G., Rios-Pohl L., Giustina E.D., Rubboli G. (2009). Encephalopathy with status epilepticus during slow sleep: “the Penelope syndrome”. Epilepsia.

[bib85] Tononi G., Cirelli C. (2003). Sleep and synaptic homeostasis: A hypothesis. Brain Research Bulletin.

[bib86] Tononi G., Cirelli C. (2006). Sleep function and synaptic homeostasis. Sleep Medicine Reviews.

[bib87] Tramoni E., Felician O., Barbeau E.J., Guedj E., Guye M., Bartolomei F. (2011). Long-term consolidation of declarative memory: Insight from temporal lobe epilepsy. Brain.

[bib88] Treves A., Rolls E.T. (1994). Computational analysis of the role of the hippocampus in memory. Hippocampus.

[bib89] Urbain C., Di Vincenzo T., Peigneux P., Van Bogaert P. (2011). Is sleep-related consolidation impaired in focal idiopathic epilepsies of childhood? A pilot study. Epilepsy & Behavior.

[bib90] Verrotti A., Filippini M., Matricardi S., Agostinelli M.F., Gobbi G. (2014). Memory impairment and benign epilepsy with centrotemporal spike (BECTS): A growing suspicion. Brain and Cognition.

[bib91] Warrington E.K., Weiskrantz L. (1978). Further analysis of the prior learning effect in amnesic patients. Neuropsychologia.

[bib92] Wilkinson H., Holdstock J.S., Baker G., Herbert A., Clague F., Downes J.J. (2012). Long-term accelerated forgetting of verbal and non-verbal information in temporal lobe epilepsy. Cortex.

[bib93] Wixted J.T. (2004). The psychology and neuroscience of forgetting. Annual Review of Psychology.

[bib94] Wixted J.T., Della Sala S. (2010). The role of retroactive interference and consolidation in everyday forgetting. Forgetting.

[bib95] Yaroush R., Sullivan M.J., Ekstrand B.R. (1971). Effect of sleep on memory. II. Differential effect of the first and second half of the night. Journal of Experimental Psychology.

[bib96] Yassa M.A., Stark C.E. (2011). Pattern separation in the hippocampus. Trends in Neurosciences.

[bib97] Zeman A.Z., Boniface S.J., Hodges J.R. (1998). Transient epileptic amnesia: A description of the clinical and neuropsychological features in 10 cases and a review of the literature. Journal of Neurology Neurosurgery and Psychiatry.

[bib98] Zeman A.Z., Butler C. (2010). Transient epileptic amnesia. Current Opinion in Neurology.

[bib99] Zeman A.Z., Butler C., Muhlert N., Milton F. (2013). Novel forms of forgetting in temporal lobe epilepsy. Epilepsy & Behavior.

