# Supplementary material for: Slow wave sleep and accelerated forgetting
Source: Cortex. 2016 Nov;84:80–9. doi: 10.1016/j.cortex.2016.08.013 (PMC5084685; doi:10.1016/j.cortex.2016.08.013)
Supplement: Supplementary file 1 [file mmc1.docx]

**Supplementary Material**

**Supplementary Methods**

*Participants*

Thirteen patients who met the diagnostic criteria for TEA, reported symptoms suggestive of ALF, and did not exhibit impairments on standard neuropsychological tests designed to measure general cognitive ability (i.e. had an IQ of at least 80, according to the Wechsler Abbreviated Scale of Intelligence (Wechsler, 1999)(Wechsler, 1999) and the National Adult Reading Scale (H. Nelson & Willison, 1991; H. E. Nelson, 1982)(H. Nelson & Willison, 1991; H. E. Nelson, 1982)) were recruited. The diagnostic criteria for TEA (taken from Zeman & Butler, 2010)(taken from Zeman & Butler, 2010) are: (1) A history of recurrent witnessed episodes of transient amnesia (2) Cognitive functions other than memory judged to be intact during typical episodes by a reliable witness (3) Evidence for a diagnosis of epilepsy based on one or more of the following: (a) Epileptiform abnormalities on electroencephalography (b) The concurrent onset of other clinical features of epilepsy (e.g. lip-smacking, olfactory hallucinations) (c) A clear-cut response to anticonvulsant therapy. Fifteen control participants were recruited by advertisement. The control participants received £120 for taking part. The patients were not offered a financial incentive for taking part, so as to avoid any possibility of exploitation. Ideally, conditions should always be matched as closely as possible between groups, but it would have been very difficult to recruit healthy control participants without compensating them for their time.

As described in Atherton et al. (2014)(2014), one control did not reach criterion on the task and did not complete the experiment. One control was excluded because his performance on the 12-hrs test was more than two standard deviations below the mean of the other participants. A further three participants were also excluded: the youngest control (to make the patient and control groups more closely matched in terms of age); a patient who repeatedly delayed the training procedure (his average response time in the training tests was more than four standard deviations greater than the mean of the included participants); and another patient was removed to allow better counterbalancing of the versions of the experiment. As stated in Atherton et al., (2014)(2014), exclusion of these three participants did not alter the significance of any of the long-term memory retention results we reported. However, if they had not been excluded, the two groups would not have been matched for age, experiment version and performance over the first 30 minutes of the experiment, and this could have confounded interpretation of the results.

Details about the eleven remaining patients are provided in Table 1. All patients were on anticonvulsant monotherapy and, in all cases but one, had been free of seizures for at least six months prior to testing. No patients reported seizures during the experiment. These eleven patients were matched in terms of age, IQ and performance on a range of standard neuropsychological tests (see Table 2) to the group of twelve remaining control participants. The control participants did not have any sleep, central nervous system or psychiatric disorders and did not complain of ALF. The participants were not shift workers, did not consume alcohol during the experiment, and had not crossed time zones in the preceding weeks.

*Task*

The details of the word-pair associates task are provided in Atherton et al. (2014)(2014). Briefly (see Figure s1), participants were trained to 60% criterion on 30 unrelated A-B word-pairs at eight am/pm and tested 30 minutes later. Twelve hours later (eight pm/am, after a night of sleep or a day of wakefulness) participants were exposed to, and then immediately tested on, interference (A-C) pairs. Ten minutes later, participants were shown the cue (A) words and asked to produce both the paired associates (B & C). Performance on the B paired associates was of primary interest; interference was introduced principally to unmask the benefit of sleep for memory (Ellenbogen, Hulbert, Jiang, & Stickgold, 2009; Ellenbogen, Hulbert, Stickgold, Dinges, & Thompson-Schill, 2006)(Ellenbogen, Hulbert, Jiang, & Stickgold, 2009; Ellenbogen, Hulbert, Stickgold, Dinges, & Thompson-Schill, 2006). Each person participated in both the sleep and wake conditions, with 24 hours in between. The order was counterbalanced across participants. Two sets of word-pairs were used so that the stimuli were novel for each condition. The order in which the stimuli were used, and the distribution of stimuli across conditions, were counterbalanced across participants.

There was an additional memory test one week later, which was performed over the telephone. The internet was used to deliver the stimuli to the participants’ own computer on the day of testing. In the event that the participant did not have access to a computer, he/she was provided with a digital photoframe, preloaded with the test stimuli, or a stimulus booklet. The word-pairs from the first condition were tested first, followed by those from the second condition. The procedure for each trial was very similar to that in the 12-hrs (A-B&C) tests, except that there was no presentation time limit - each cue (A) word was presented until the participant had made an attempt to produce both paired associates - and no stimuli (such as fixation crosses, as described in Appendix A of Atherton et al., 2014)(such as fixation crosses, as described in Appendix A of Atherton et al., 2014) intervened between the cue words.

*Spindle detection*

We followed the widespread threefold thresholding procedure selecting oscillatory transients by their frequency, amplitude and duration complemented by a proximity criterion that, if met, merges adjacent events. The algorithm was applied separately on each of the derivations re-referenced to the contralateral mastoid electrodes. The signal was digitized at a sampling rate of Fs = 256Hz, bandpass filtered using an FIR filter of order 603 (for Fs=256Hz) parametrized using the Remez exchange algorithm (McClellan & Parks, 1973) (passband: 11.3-15.7Hz, stopband: 10-17Hz, stopband attenuation: 1.122 x 10^(-5), passband ripple: 0.0575, density factor: 20). All subsequent steps were carried out on the filtered signal.

The root mean square (RMS) signal was calculated by using a 0.12 seconds moving window with 1/Fs long time shifts, i.e., with the same time resolution as the original signal. This RMS signal was then smoothed by applying a 1.2 seconds long Hanning window.

The amplitude threshold was set to the 83rd percentile of the distribution of the so obtained RMS amplitude extracted from NREM2 stages.

Subsequent segments with RMS signal exceeding the threshold were fused (i.e., associated with the same spindle) if closer to one another than 1 second and if the duration of the resulting segment remained short of 3 seconds. In order to make the procedure unambiguous the rule was applied recursively starting from the closest pair and fusing a single pair at the time until no more mergeable pairs were found.

Eventually, segments with duration within the 0.4-3.0 seconds interval were labelled as spindles.

**Supplementary Results.**

The results of the main analysis from Atherton et al., (2014)(2014) - a mixed-effects ANOVA with A-B pair performance as the dependent variable, sleep condition (two levels: sleep and wake) and retrieval time point (three levels: final training test, 30-mins test and 12-hrs test) as the within-subjects factors and group as the between-subjects factor – are reproduced here (see Table s3 and Figure s2a).

While there were no significant group differences in A-B learning (score on the first training test or trials to criterion) or A-C performance (see Table 3), the patients demonstrated ALF (there was an interaction between retrieval time point and group, F_(1.34,28.15)_ = 6.33, p=0.012): they performed significantly more poorly than the controls on the 12-hrs test (estimated marginal means (EMMs) ±SEMs: 13.55 ±1.01 and 17.50 ±0.97, respectively, p= 0.01), but not on the final training test (EMMs ±SEMs: 22.00 ±0.68 and 22.54 ±0.65) or the 30-mins test (EMMs ±SEMs: 20.00 ±0.76 and 21.33 ±0.73). There was a benefit of sleep for memory retention (there was an interaction between sleep condition and retrieval time-point, F_(2,42)_ = 20.15, p<0.001): participants performed significantly better in the sleep condition than the wake condition on the 12-hrs test (EMMs ±SEMs: 16.90 ±0.75 and 14.14 ±0.85, p=0.002), but not on the final training test (EMMs ±SEMs: 22.10 ±0.51 and 22.45 ±0.68) or the 30-mins test (EMMs ±SEMs: 20.52 ±0.64 and 20.77 ±0.71). The benefit of sleep for memory retention was no smaller in magnitude for the patients than the controls (no significant interaction between sleep condition, retrieval time point and group).

The data from the one week test are presented in Figure s2b.

*Sleep-behaviour correlations with excluded participants added*

When the three controls and two patients who had been excluded (as described in the Participants section) were included in the sleep-behaviour correlation analyses, the pattern of the results remained the same:

The patients showed a significant negative correlation between the percentage of SWS and the benefit of sleep for memory retention over twelve hours (r = -0.691, p = 0.009). The controls did not show this negative correlation; their Pearson correlation coefficient was positive and non-significant (r = 0.362, p=0.20). A Fisher’s transformation analysis revealed that the correlation was significantly different in the two groups (z = -2.81, p<0.01).

The controls showed a significant positive correlation between the percentage of SWS and the benefit of post-learning sleep for memory retention over one week (r = 0.602, p = 0.023). The patients did not show this positive correlation. The patients’ Pearson correlation coefficient was negative and non-significant (r = -0.332, p=0.27). The Fisher’s transformation analysis revealed that the correlation was significantly different in the two groups (z =-2.38, p<0.05).

*Sleep-behaviour correlations for sleep stages other than SWS*

Two-tailed Pearson correlation tests investigating the relationship between the percentage of REM sleep and the benefit of sleep for memory retention produced no significant results (see Figure s3).

The patients showed a significant positive correlation between the percentage of NREM2 and the benefit of sleep for memory retention over twelve hours (r = 0.67, p = 0.024, see Figure s3). The controls did not show this positive correlation; their Pearson correlation coefficient was non-significant (r =,-0.15 p = 0.64). A Fisher’s transformation analysis revealed that the correlation was significantly different in the two groups (z = 2.15, p = 0.032).

Sleep spindles

Figure s4 plots the benefit of sleep for memory retention against spindle incidence during SWS.

Sleep spindles are prevalent in NREM2 and so, while NREM2 was not of a priori interest, data on sleep spindle incidence in NREM2 are presented below.

Independent samples t-tests revealed no significant difference between patients and controls in sleep spindle incidence in NREM2 (5.70 ±0.29 and 5.81 ±0.43 spindles per minute, p = 0.84).

Two-tailed Pearson correlation tests investigating the relationship between the incidence of spindles in NREM2 and the benefit of sleep for memory retention in each group (see Figure s5) produced no significant results (all ps>0.27).

**Supplementary References**

Atherton, K. E., Nobre, A. C., Zeman, A. Z., & Butler, C. R. (2014). Sleep-dependent memory consolidation and accelerated forgetting. *Cortex, 54*, 92-105. doi:10.1016/j.cortex.2014.02.009

Delis, D. C., Kaplan, E., & Kramer, J. H. (2001). *Delis-Kaplan Executive Function System (D-KEFS)*. San Antonio, TX: Psychological Corporation.

Ellenbogen, J. M., Hulbert, J. C., Jiang, Y., & Stickgold, R. (2009). The sleeping brain's influence on verbal memory: boosting resistance to interference. *PLoS One, 4*(1), e4117. doi:10.1371/journal.pone.0004117

Ellenbogen, J. M., Hulbert, J. C., Stickgold, R., Dinges, D. F., & Thompson-Schill, S. L. (2006). Interfering with theories of sleep and memory: sleep, declarative memory, and associative interference. *Curr Biol, 16*(13), 1290-1294. doi:10.1016/j.cub.2006.05.024

McClellan, J., & Parks, T. (1973). A unified approach to the design of optimum FIR linear-phase digital filters. *IEEE Transactions on Circuit Theory, 20*(6), 697-701.

McKenna, P., & Warrington, E. K. (1980). Testing for nominal dysphasia. *Journal of Neurology, Neurosurgery & Psychiatry, 43*(9), 781-788.

Nelson, H., & Willison, J. (1991). The revised national adult reading test–test manual. *Windsor: NFER-Nelson*.

Nelson, H. E. (1982). *The National Adult Reading Test (NART): Test manual, 1-13*. Windsor: NFER-Nelson.

Rey, A. (1941). L’examen psychologique dans les cas d’encephalopathie traumatique. *Arch. Psychol., 28*, 286-340.

Warrington, E. K. (1984). *The recognition memory test*. Windsor, United Kingdom: NFER-Nelson.

Wechsler, D. (1955). *Manual for the Wechsler Adult Intelligence Scale*. New York: Psychological Corporation.

Wechsler, D. (1997). *Wechsler memory scale III*. San Antonio, TX: Psychological Corporation.

Wechsler, D. (1999). *Wechsler abbreviated scale of intelligence*. San Antonio, TX: Psychological Corporation.

Zeman, A., & Butler, C. (2010). Transient epileptic amnesia. *Curr Opin Neurol, 23*(6), 610-616. doi:10.1097/WCO.0b013e32834027db

Zigmond, A. S., & Snaith, R. P. (1983). The hospital anxiety and depression scale. *Acta psychiatrica scandinavica, 67*(6), 361-370.
