# Supplementary figures and images for: Slow wave sleep and accelerated forgetting
Source: Cortex. 2016 Nov;84:80–9. doi: 10.1016/j.cortex.2016.08.013 (PMC5084685; doi:10.1016/j.cortex.2016.08.013)

## Slide 1
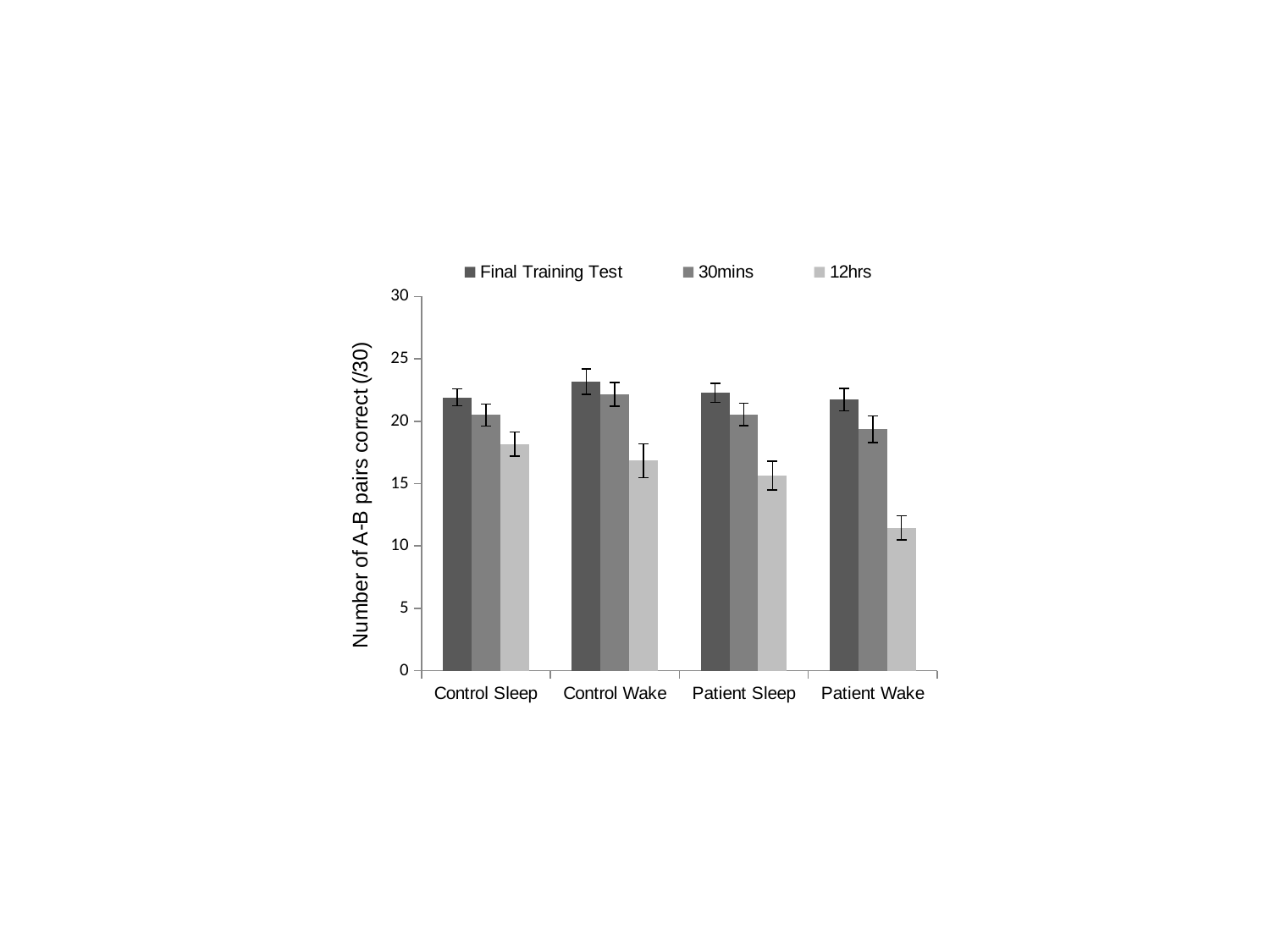

### Chart
| Category | | | |
|---|---|---|---|
| Control Sleep | 21.91666666666667 | 20.5 | 18.16666666666667 |
| Control Wake | 23.16666666666667 | 22.16666666666667 | 16.8333333333331 |
| Patient Sleep | 22.27272727272688 | 20.54545454545455 | 15.63636363636364 |
| Patient Wake | 21.72727272727273 | 19.36363636363628 | 11.45454545454552 |

Supplement: Fig. S2a — A–B pair performance in the final training test, the 30-min test and the 12-h test in the sleep and wake conditions of the word-pair associates task, in people with TEA-associated ALF and control participants, taken from Atherton et al. (2014). Error bars represent standard errors of the mean. [file mmc2.pptx]

## Slide 1
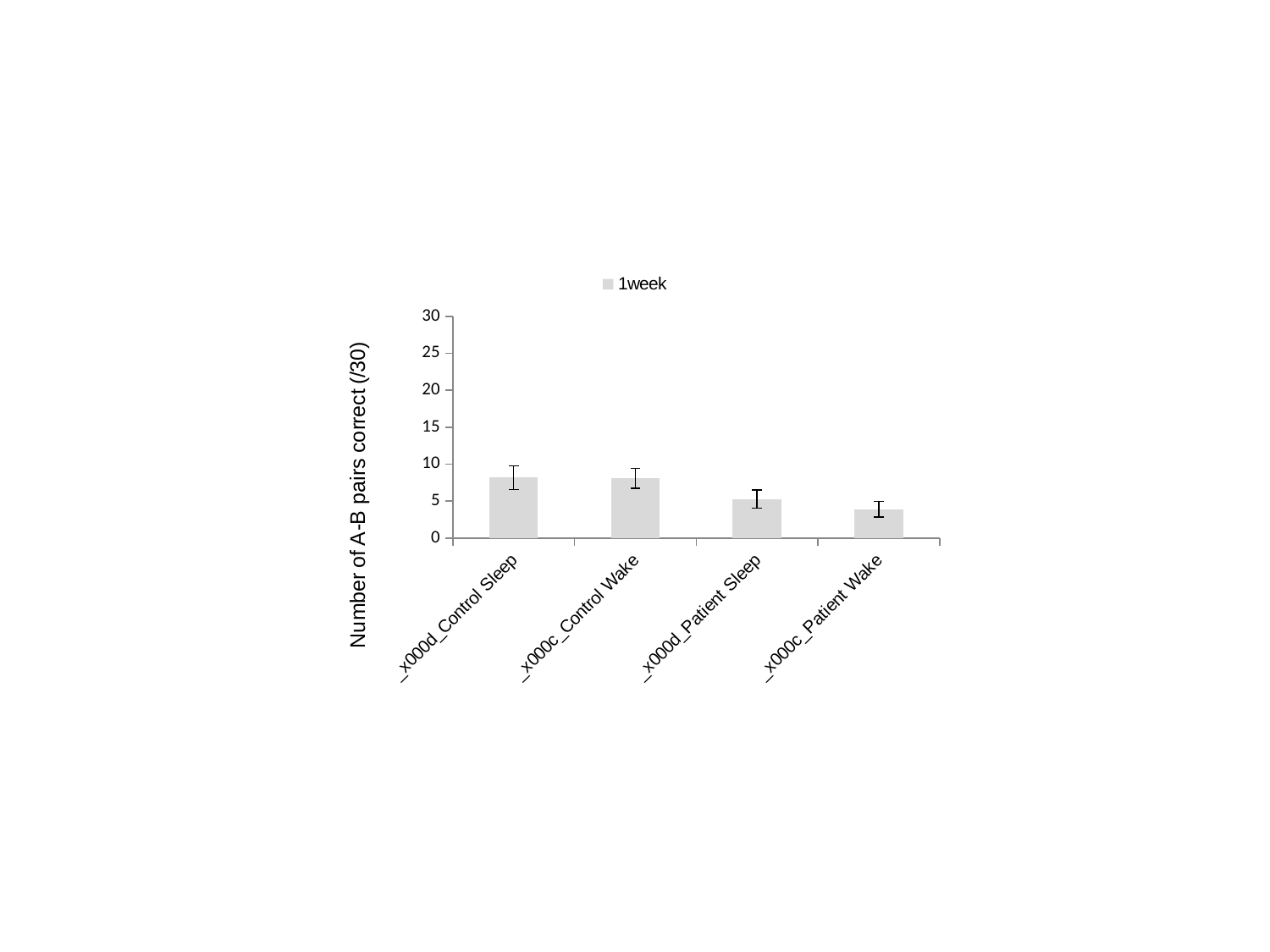

### Chart
| Category | |
|---|---|
| _x000d_Control Sleep | 8.16666666666667 |
| _x000c_Control Wake | 8.083333333333332 |
| _x000d_Patient Sleep | 5.272727272727272 |
| _x000c_Patient Wake | 3.909090909090909 |

Supplement: Fig. S2b — A–B pair performance in the 1-week test in the sleep and wake conditions of the word-pair associates task, in people with TEA-associated ALF and control participants. Error bars represent standard errors of the mean. [file mmc3.pptx]
