# Supplementary material for: Slow wave sleep and accelerated forgetting
Source: Cortex. 2016 Nov;84:80–9. doi: 10.1016/j.cortex.2016.08.013 (PMC5084685; doi:10.1016/j.cortex.2016.08.013)
Supplement: Fig. S3 — The benefit of post-learning sleep for memory retention over twelve hours (top charts) and one week (bottom charts) plotted against the percentage of total sleep time spent in REM sleep (left charts) and NREM2 sleep (right charts) during the sleep condition night. The lines of best fit and R2 values are displayed for the significant correlations. The patients showed a significant positive correlation between NREM2 (%) and the benefit of post-learning sleep for memory retention over twelve hours. This correlation was not seen in the controls and the correlation significantly differed between the two groups. [file mmc4.pptx]

## Slide 1
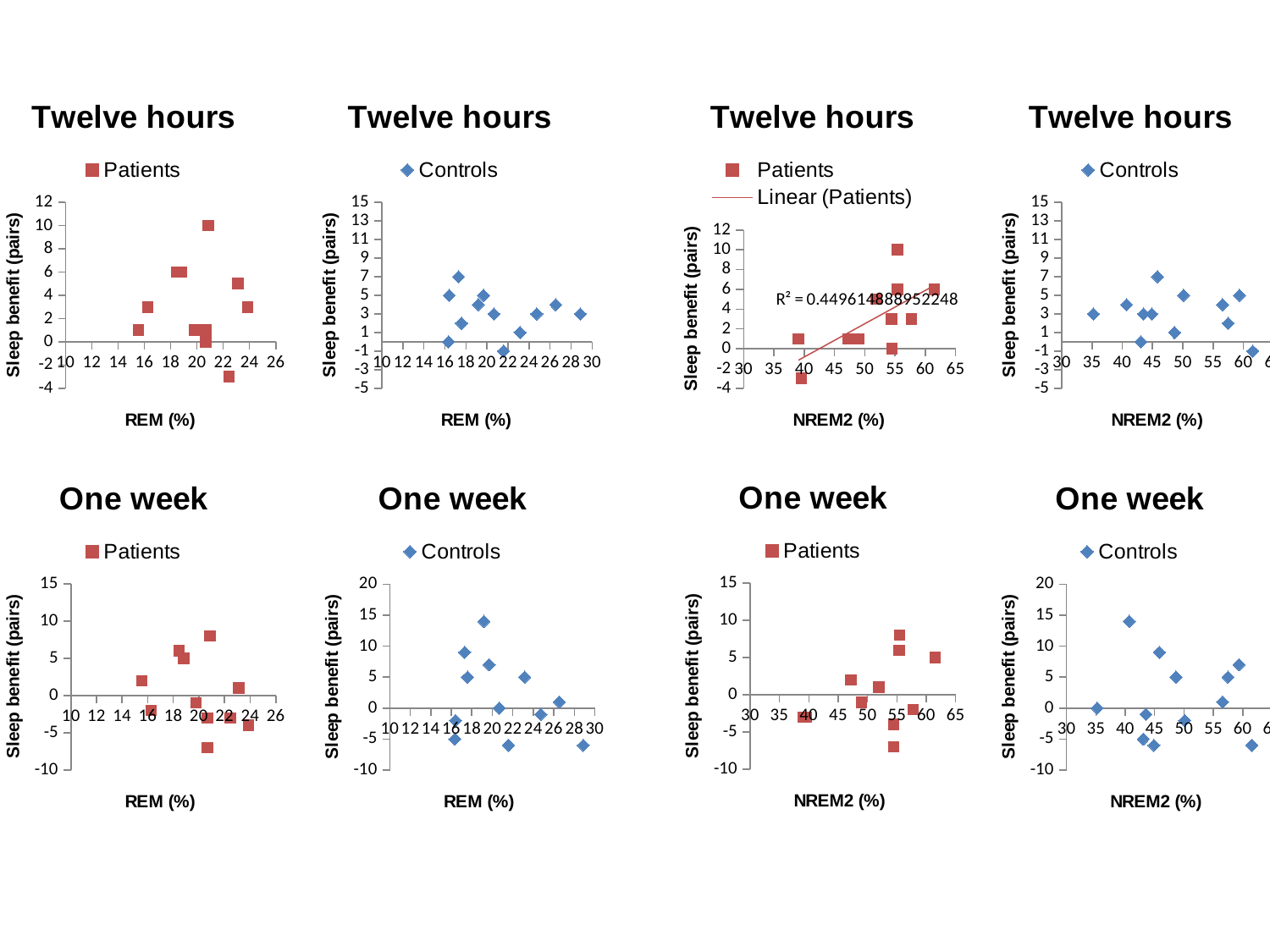

### Chart: Twelve hours
| Category | |
|---|---|
### Chart: Twelve hours
| Category | |
|---|---|
### Chart: Twelve hours
| Category | |
|---|---|
### Chart: Twelve hours
| Category | |
|---|---|
### Chart: One week
| Category | |
|---|---|
### Chart: One week
| Category | |
|---|---|
### Chart: One week
| Category | |
|---|---|
### Chart: One week
| Category | |
|---|---|
