# Supplementary material for: Slow wave sleep and accelerated forgetting
Source: Cortex. 2016 Nov;84:80–9. doi: 10.1016/j.cortex.2016.08.013 (PMC5084685; doi:10.1016/j.cortex.2016.08.013)
Supplement: Fig. S5 — The benefit of post-learning sleep for memory retention over twelve hours (top charts) and one week (bottom charts) plotted against the spindle incidence in NREM2 (the number of spindles in NREM2/the total number of artefact free NREM2 min) during the sleep condition night. There were no significant results. [file mmc6.pptx]

## Slide 1
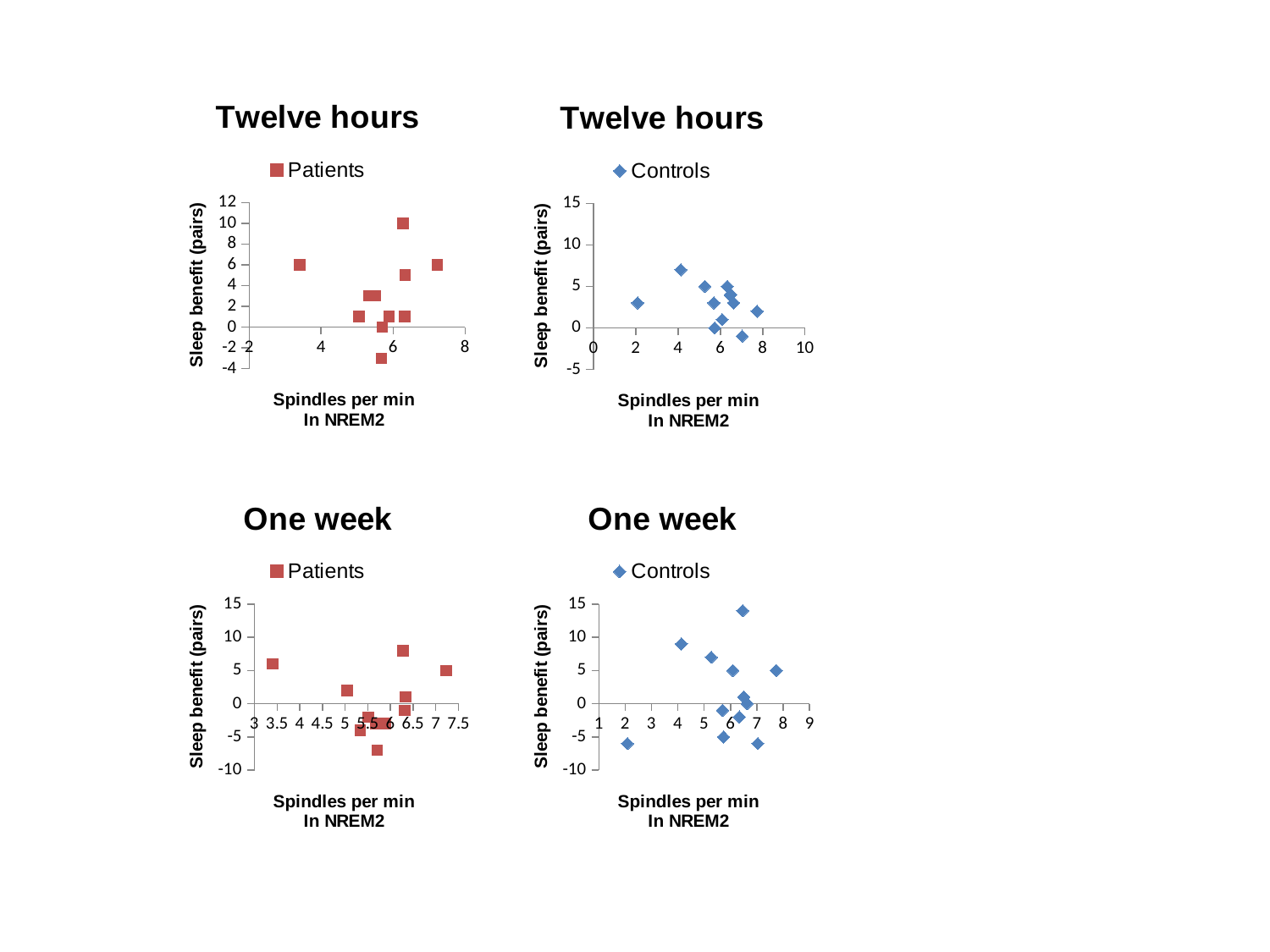

### Chart: Twelve hours
| Category | |
|---|---|
### Chart: Twelve hours
| Category | |
|---|---|
### Chart: One week
| Category | |
|---|---|
### Chart: One week
| Category | |
|---|---|
