# Supplementary material for: Slow wave sleep and accelerated forgetting
Source: Cortex. 2016 Nov;84:80–9. doi: 10.1016/j.cortex.2016.08.013 (PMC5084685; doi:10.1016/j.cortex.2016.08.013)
Supplement: Table S1 — TEA patient information, adapted from Atherton et al. (2014). AEDs = Anti-Epileptic Drugs that the patients were taking when they took part in the sleep experiment, LEV = Levetiracetam, CBZ = Carbamazepine, LTG = Lamotrigine, SVP = Sodium Valproate. [file mmc7.docx]

| Gender | Age | Age at | AEDs (type & | Evidence for a diagnosis of epilepsy | | | MRI |
| --- | --- | --- | --- | --- | --- | --- | --- |
|  |  | **onset** | mg per day) | EEG | Other Features | Treatment Response |  |
| M | 70 | 66 | LEV 2000 | Non-specific bilateral temporal lobe slowing | Oroalimentary automatisms, gustatory hallucinations | Complete | Normal |
|  |  |  |  |  |  |  |  |
| M | 62 | 54 | LTG 200 | L temporal epileptiform | Oroalimentary automatisms | Complete | Normal |
|  |  |  |  |  |  |  |  |
| M | 68 | 65 | CBZ 400 | Non-specific L temporal slowing | No | Complete | Normal |
|  |  |  |  |  |  |  |  |
| M | 72 | 71 | LTG 200 | Non-specific R temporal slowing | Déjà vu | Complete | Bilateral high T2 signal in hippocampus |
| M | 68 | 62 | CBZ 400 | Bilateral (L more marked) mid-anterior temporal sharp and slow-wave epileptiform | Oroalimentary automatisms; brief unresponsiveness | Complete | Normal |
|  |  |  |  |  |  |  |  |
| M | 76 | 65 | LTG 100 | Non-specific bilateral temporal slowing | Olfactory hallucinations; oroalimentary automatisms | Complete | Normal |
|  |  |  |  |  |  |  |  |
| M | 66 | 56 | LTG 150 | Normal | Olfactory hallucinations; brief unresponsiveness | Complete | Normal |
|  |  |  |  |  |  |  |  |
| M | 64 | 61 | SVP 800 | Bilateral temporal epileptiform | Olfactory hallucinations; oroalimentary automatisms | Complete | Normal |
|  |  |  |  |  |  |  |  |
| M | 63 | 59 | LEV 500 | Normal | No | Partial | Normal |
|  |  |  |  |  |  |  |  |
| M | 76 | 73 | SVP 400 | Normal | Oroalimentary automatisms; brief unresponsiveness | Complete | Small area of right frontal encephalomalacia |
|  |  |  |  |  |  |  |  |
| F | 60 | 55 | CBZ 300 | Non-specific bilateral temporal slowing | Brief unresponsiveness | Complete | Normal |
