# Supplementary material for: Slow wave sleep and accelerated forgetting
Source: Cortex. 2016 Nov;84:80–9. doi: 10.1016/j.cortex.2016.08.013 (PMC5084685; doi:10.1016/j.cortex.2016.08.013)
Supplement: Table S2 — Participant information, adapted from Atherton et al. (2014). Means with SEMs in brackets. Patients and controls did not significantly differ in terms of age, years of full-time education or test scores (ps > .05). aNART (Nelson, 1982; Nelson & Willison, 1991); bWAIS = Wechsler Abbreviated Intelligence Scale (Wechsler, 1955); cWASI = Wechsler Abbreviated Scale of Intelligence (Wechsler, 1999); dWMS-III = Wechsler Memory Scale-III (Wechsler, 1997); eRey-Osterrieth complex figure (Rey, 1941); fRMT (Warrington, 1984); gGNT (McKenna & Warrington, 1980); hDKEFS = Delis-Kaplan Executive Function System (Delis et al., 2001); iHADS (Zigmond & Snaith, 1983). [file mmc8.docx]

|  | TEA Patients | Controls |
| --- | --- | --- |
| N | 11 | 12 |
| Gender | 1 female | 5 females |
| Duration of epilepsy (months) | 69.55(±10.45) | n/a |
| Time between neuropsychological tests and experiment (months) | -7.36(±3.32) | 3.17(±1.09) |
|  |  |  |
| Age | 67.73(±1.63) | 63.50(±1.44) |
| Years of full-time education | 13.45(±2.84) | 13.04(±3.44) |
|  |  |  |
| *IQ tests* |  |  |
| Predicted WAIS^b^ verbal IQ from NART errors | 117.91(±1.89) | 118.67(±1.87) |
| WASI verbal IQ | 116.27(±2.22) | 120.83(±3.05) |
| WASI performance IQ | 120.18(±2.73) | 117.00(±3.30) |
| WASI full scale-4subtests IQ | 120.36(±1.97) | 121.42(±3.01) |
| *Anterograde memory* |  |  |
| WMS-III^d^ Logical memory story: Immediate recall (max 25) | 14.73(±0.82) | 17.50(±1.31) |
| WMS-III Logical memory story: Delayed recall (30mins) (max 25) | 12.18(±1.30) | 14.75(±1.41) |
| WMS-III Logical memory story: Delayed recognition (30mins) (max 15) | 13.18(±0.38) | 13.00(±0.41) |
| Rey-Osterrieth Complex figure^e^: Copy (max 36) | 33.50(±0.93) | 32.38(±0.51) |
| Rey-Osterrieth Complex figure: Delayed recall (30mins) (max 36) | 16.86(±1.62) | 18.25(±1.08) |
| Recognition Memory Test (RMT)^f^: Words (max 50) | 46.36(±0.81) | 47.50(±0.87) |
| Recognition Memory Test (RMT): Faces (max 50) | 41.72(±1.40) | 44.83(±0.81) |
|  |  |  |
| *Semantic memory* |  |  |
| Graded Naming Test (GNT)^g^ (max 30) | 24.27(±1.18) | 25.08(±0.63) |
|  |  |  |
| *Executive function* |  |  |
| DKEFS^h^ verbal fluency letters (No. of words in 3mins) | 46.73(±2.76) | 48.75(±4.74) |
| DKEFS verbal fluency categories (No. of words in 1min) | 39.45(±3.00) | 46.33(±2.76) |
| DKEFS verbal fluency switching (No. of words in 1min) | 13.45(±0.73) | 15.75(±0.92) |
| DKEFS trails 4 (switching) (seconds to complete) | 87.57(±9.99) | 84.38(±12.99) |
| WMS-III Digit span forwards (max 16) | 12.36(±0.79) | 11.00(±0.65) |
| WMS-III Digit span backwards (max 14) | 9.27(±1.05) | 7.67(±0.76) |
|  |  |  |
| *Anxiety and Depression scores* |  |  |
| Hospital Anxiety and Depression Scale (HADS)^i^ anxiety (max 21) | 6.36(±1.19) | 5.33(±0.71) |
| Hospital Anxiety and Depression Scale (HADS) depression (max 21) | 4.36(±0.87) | 2.58(±0.80) |
