# Supplementary material for: Slow wave sleep and accelerated forgetting
Source: Cortex. 2016 Nov;84:80–9. doi: 10.1016/j.cortex.2016.08.013 (PMC5084685; doi:10.1016/j.cortex.2016.08.013)
Supplement: Table S3 — Performance on the word-pair associates task, adapted from Atherton et al. (2014). Means with SEMs in brackets. The three A–B word-pair tests that are plotted in Fig. 2a are in boldface. [file mmc9.docx]

|  | Controls | | Patients | |
| --- | --- | --- | --- | --- |
|  | Sleep | Wake | Sleep | Wake |
| 1st training test score  (/30) (A-B) | 10.17(±2.39) | 10.75(±1.75) | 8.45(±1.83) | 7.91(±1.21) |
|  |  |  |  |  |
| No. trials to criterion  (A-B) | 2.00(±0.21) | 2.50(0.26) | 2.82(±0.48) | 2.27(±0.14) |
|  |  |  |  |  |
| **Final training test score**  **(/30)** (A-B) | **21.92(±0.68)** | **23.17(±1.01)** | **22.27(±0.75)** | **21.72(±0.91)** |
|  |  |  |  |  |
| **30-mins test score**  **(/30)** (A-B) | **20.50(±0.89)** | **22.17(±0.95)** | **20.55(±0.91)** | **19.36(±1.06)** |
|  |  |  |  |  |
| Immediate interference pair score (/30) (A-C) | 8.50(±2.04) | 9.67(±1.94) | 10.00(±1.68) | 8.36(±1.01) |
|  |  |  |  |  |
| **12-hrs test score**  **(/30)** (A-B) | **18.17(±0.98)** | **16.83(±1.36)** | **15.64(±1.15)** | **11.45(±0.98)** |
|  |  |  |  |  |
| Interference pair score  (/30) (A-C) | 8.17(±1.85) | 9.42(±1.94) | 7.91(±1.68) | 8.09(±1.25) |
|  |  |  |  |  |
| 1-week test score  (/30) (A-B) | 8.17(±1.63) | 8.08(±1.33) | 5.27(±1.26) | 3.91(±1.05) |
|  |  |  |  |  |
| 1-week interference pair score (/30) (A-C) | 1.17(±0.46) | 2.41(±0.70) | 0.82(±0.38) | 0.92(±0.46) |
